# Pairing virtual reality with dynamic posturography serves to differentiate between patients experiencing visual vertigo

**DOI:** 10.1186/1743-0003-4-24

**Published:** 2007-07-09

**Authors:** Emily A Keshner, Jefferson Streepey, Yasin Dhaher, Timothy Hain

**Affiliations:** 1 Sensory Motor Performance Program, Rehabilitation Institute of Chicago, Room 1406, 345 East Superior St., Chicago, IL 60611 USA; 2 Department of Physical Medicine and Rehabilitation, Feinberg School of Medicine, Northwestern University, 345 East Superior St., Chicago, IL 60611 USA; 3 Department of Physical Therapy and Human Movement Science, Feinberg School of Medicine, Northwestern University, 645 N. Michigan Ave., Chicago, IL 60611 USA; 4 Biomedical Engineering Department, Northwestern University, 2145 Sheridan Road, Evanston, IL 60208-3107 USA; 5 Dept. of Physical Therapy, College of Health Professions, Temple University, Jones Hall 600, 3307 Broad St., Philadelphia PA 19140 USA

## Abstract

**Background:**

To determine if increased visual dependence can be quantified through its impact on automatic postural responses, we have measured the combined effect on the latencies and magnitudes of postural response kinematics of transient optic flow in the pitch plane with platform rotations and translations.

**Methods:**

Six healthy (29–31 yrs) and 4 visually sensitive (27–57 yrs) subjects stood on a platform rotated (6 deg of dorsiflexion at 30 deg/sec) or translated (5 cm at 5 deg/sec) for 200 msec. Subjects either had eyes closed or viewed an immersive, stereo, wide field of view virtual environment (scene) moved in upward pitch for a 200 msec period for three 30 sec trials at 5 velocities. RMS values and peak velocities of head, trunk, and head with respect to trunk were calculated. EMG responses of 6 trunk and lower limb muscles were collected and latencies and magnitudes of responses determined.

**Results:**

No effect of visual velocity was observed in EMG response latencies and magnitudes. Healthy subjects exhibited significant effects (*p *< 0.05) of visual field velocity on peak angular velocities of the head. Head and trunk velocities and RMS values of visually sensitive subjects were significantly larger than healthy subjects (*p *< 0.05), but their responses were not modulated by visual field velocity. When examined individually, patients with no history of vestibular disorder demonstrated exceedingly large head velocities; patients with a history of vestibular disorder exhibited head velocities that fell within the bandwidth of healthy subjects.

**Conclusion:**

Differentiation of postural kinematics in visually sensitive subjects when exposed to the combined perturbations suggests that virtual reality technology could be useful for differential diagnosis and specifically designed interventions for individuals whose chief complaint is sensitivity to visual motion.

## Background

Visual vertigo is defined as dizziness provoked by visual environments with full field of view repetitive or moving visual patterns [1]. For example, patients with visual vertigo report discomfort in supermarkets and when viewing crowds and traffic. Visual vertigo is present in many patients with a history of a peripheral vestibular disorder. There is also a subset of patients who have no history of vestibular disorder and who test negative for vestibular deficit on traditional clinical tests, but who still report severe reactions to visual motion. In fact, dizziness and vertigo, dizziness, and unsteadiness are frequently encountered symptoms in ENT, neurology, and general practice [2].

It has been suggested that patients with visual vertigo benefit from exercises involving visuo-vestibular conflict [3] which assumes that their visual sensitivity emerges from an inability to adapt to a conflict between visual and vestibular inputs. If this were the case, we would expect these individuals to demonstrate impaired postural control as well. Increased visual dependence has been demonstrated to produce an altered postural sway during quiet stance when placed in a disorienting visual environment [4], however, visual inputs are not considered to be responsible for the generation of healthy automatic postural reactions [5,6]. Thus, we hypothesized that increased visual sensitivity would emerge as changes in the angular velocity of the automatic postural reactions that linearly reflected changes in the angular velocity of the visuospatial environment.

## Methods

### Subjects

Six healthy young adult (29–31 yrs) subjects (HS) and four subjects (VS) diagnosed as having visual vertigo (27–57 yrs) participated in these experiments. Healthy subjects were free from musculoskeletal and neurological disorders. All subjects responded to the Situational Characteristics Questionnaire and the Dizziness Handicap Inventory. Subjects provided written consent in accordance with the Institutional Review Board, Feinberg School of Medicine, Northwestern University. One VS subject (VS1) developed profound sensitivity to visual stimuli with no known etiology. ENG and MRI of the brain were normal as was her general neurological examination except for extremely poor smooth visual pursuit. VS2 experienced dizziness when standing up, performing rapid head movements, walking in a dark room, and in busy sensory environments after being treated with an intravenous antibiotic in 2003. General neurological examination, ENG testing, and rotatory chair testing was normal. The dynamic illegible 'E' test [7] in which subjects attempt to read a Snellen chart while their head is rotated in yaw was reduced by 4 lines. Both VS3 and VS4 had migraine associated vertigo and developed visual sensitivity 2 years post onset of BPPV. In response to the questionnaires, all VS subjects report being bothered by elevator and motor vehicle travel, and by complex visual environments such as grocery stores.

### Procedures

Subjects stood barefoot with feet in parallel and shoulder-width apart on a platform (Neurocom, Inc.) that was dorsiflexion rotated 6 deg at 30 deg/sec for 200 ms and held in that position for 800 ms. Additionally, 9 catch trials of anterior translation (5 cm at 5 deg/sec for 1 sec) of the platform were randomly inserted into the experimental period. The platform was placed within a single-wall virtual reality system. The screen in our system consisted of back projection material measuring 2.6 m × 3.2 m. An Electrohome Marquis 8500 projector throws a full-color stereo workstation field (1024 × 768 stereo) at 120 Hz onto the screen that fills the horizontal and vertical field of view. A dual processor PC with a nVidia Quadro [FX3000G] graphics card created the imagery projected onto the wall using the CAVELib (VRCO, Inc) VR library. The field sequential stereo images generated by the PC were separated into right and left eye images using liquid crystal stereo shutter glasses worn by the subject (Crystal Eyes, StereoGraphics Inc.) which limit the horizontal and vertical field of view to 100° and 55° of binocular vision. The projected image (scene) consisted of a 30.5 m wide by 6.1 m high by 30.5 m deep room containing round columns with patterned rugs and a painted ceiling. [See Additional File 1 to view a movie of a subject standing on the platform in front of the virtual environment and wearing the shutter glasses].

Subjects either closed their eyes (DARK) or viewed the visual scene as it rotated upward in pitch at 5 velocities: 0 (NV), 30, 45, 60, and 90 deg/sec for a period of 200 ms. After a 10 sec period of quiet stance, the platform and visual scene simultaneously pitched upward. The catch trials were also performed with four of these visual conditions (DARK, NV, 30, and 60 deg/sec). In the NV (natural vision) condition, the correct scene perspective was continuously updated by motion capture markers placed on the shutter glasses to provide feedback about position of the head in space with a total display system delay of 25 ms. Three 30 sec trials were presented for each visual condition with platform and visual stimulus motion beginning after 10 sec of quiet stance.

### Data Analysis

Three-dimensional kinematic data was collected from reflective markers at 120 Hz using a six-camera Motion Analysis system (Motion Analysis, Inc.) and low-pass filtered at 4 Hz using a 4^th ^order Butterworth digital filter. Peak angular displacement was derived using commercial software (Kintrak, Motion Analysis, Inc). Root mean square values were calculated over a 250 ms period (automatic reactions) following onset of platform motion, and over a 1000 ms (recovery) period for the head in space (from the marker placed above the left temple on the back of the head), trunk in space (from the marker on T1 and a virtual marker consisting of the average location of the right and left hip markers), and the head with respect to trunk. These data were averaged across the 3 trials of each visual condition. Statistical comparisons were performed within subjects and across scene velocities with the Wilcoxon matched pairs test. HS and VS subjects were compared with the Wilcoxon signed ranks test.

Electromyographic (EMG) responses were recorded for the left soleus (SOL), tibialis anterior (TA), vastus medialis (VM), biceps femoris (BF), erector spinae (ES), and abdominal (ABD) muscles with pairs of active parallel bar electrodes (Delsys, Inc.). Raw EMG data were filtered through identical analog low-pass filters (8-pole, Bessel) with a 500 Hz cutoff frequency prior to analog to digital conversion. Rectification and low pass filtering was done digitally. EMG data were examined for individual trials to determine the latency of the first peak of the EMG response, defined as the first peak rising 3 standard deviations above resting level activity, following onset of platform motion. EMG response latencies were not affected by the different visual velocities, thus they were averaged across visual velocities (Table 1). Area under the curve of each EMG signal was calculated for 6 periods following onset of the response: 20–50 ms, 40–100 ms, 80–120 ms, 120–220 ms, 240–340 ms, and 350–700 ms.

**Table 1:** Mean ± SD EMG Onset Latency (ms) Across All Scene Conditions

	**HS**	**VS1, 3, 4**	**VS2**
SOL	31 ± 24	33 ± 15	87 ± 16
TA	41 ± 27	42 ± 26	137 ± 11
BF	20 ± 4	35 ± 3	108 ± 18
VM	18 ± 7	23 ± 3	236 ± 76
ES	19 ± 4	28 ± 10	220 ± 35
ABD	19 ± 3	40 ± 23	164 ± 67

We also described 3-dimensional motion of head with respect to trunk during the 9 anterior translations of the platform. To do this, we used a joint coordinate system representation [8]. The first rotation was flexion-extension (pitch) through an angle *θ*_*f*/*e *_about the head medial-lateral axis (*y*-axis) followed by rotation (yaw) through an angle *θ*_*int*/*ext *_about the superior/inferior axis of the trunk (*z*-axis). The last rotation was the lateral bending (roll) *θ*_*b *_which takes place about the floating axis obtained as a result of the cross product of the two axes of rotation.

## Results

### EMG response latencies and magnitudes

There was no significant effect of visual velocity on latency of the muscle EMG responses. All of the imposed perturbations (i.e., dorsiflexion rotations of the platform, anterior translations of the platform, and upward pitch rotations of the visual scene) were expected to generate an initial posterior sway response. In the healthy subjects, the earliest muscle responses occurred in the trunk and hip muscles (Table 1) suggesting that they were exhibiting a hip strategy response [9] for stabilization. VS1, VS3, and VS4 exhibited their earliest responses in the trunk and thigh. VS2 exhibited longer latencies of EMG onset than the other subjects (Table 1). In the HS, EMG response magnitudes increased in SOL, BF, ES, and ABD in the recovery portion of the response (350–1000 ms after the platform rotation). There was a trend of increasing magnitude with increasing visual velocity although this was not significant. VS1 demonstrated a similar pattern with increased ABD activity throughout the trial. VS2 exhibited the greatest response magnitude in BF when the visual scene was moved in pitch.

### Head and trunk kinematics

**Figure 1:**
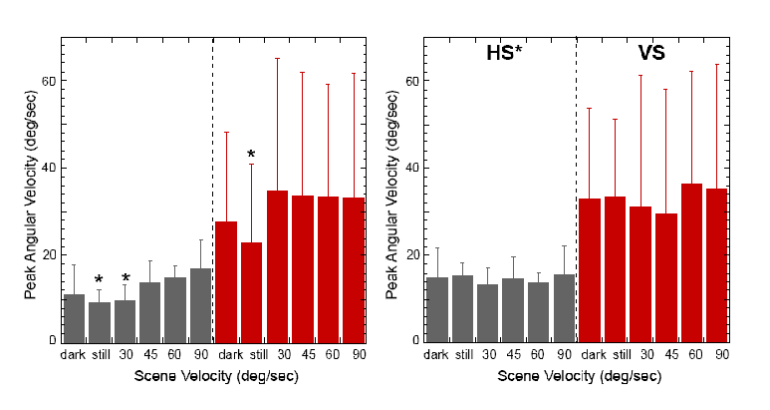
(***Left***). Average peak angular velocity of the head in space in the first 250 ms following stimulus onset at each velocity of the scene for the HS (grey) and the VS (red) subjects. Starred values are significantly less than other scene velocities (*p *< 0.05). (***Right***) Average peak angular velocity of the trunk in space across the 1000 ms period of the trial at each velocity of the scene for the HS (grey) and the VS (red) subjects. Starred values are significantly less than VS subjects (*p *< 0.05).

The most dramatic effect in VS subjects was RMS values of the head in space that were significantly larger across the period of the trial than in HS for all but the DARK condition (*p *< 0.05). Both HS and VS subjects had significantly smaller head velocities during NV than with all other scene velocities (*p *< 0.05) in the first 250 ms of the trial (Fig. 1). But only HS subjects were able to maintain this significantly smaller head velocity when the scene velocity was equal to platform velocity. Trunk in space also exhibited significantly greater RMS values in VS than HS in the first 250 ms of the trial as well as higher velocities across the trial period (*p *< 0.005) (Fig. 1).

**Figure 2:**
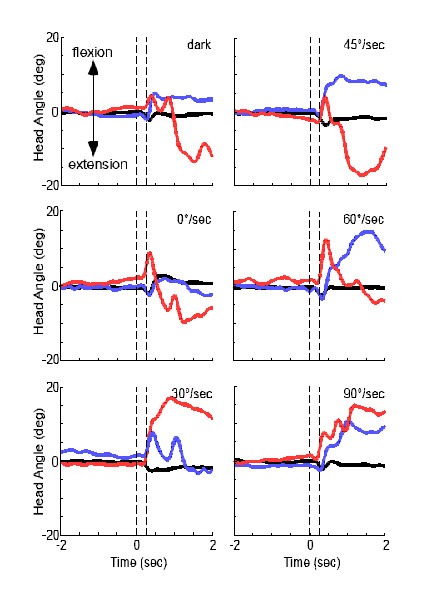
Angular excursion of the head in space for 2 sec before and 2 sec after onset of the platform and scene motion. Dashed vertical lines indicate the 200 msec period between onset and termination of the platform and visual scene disturbances. The first trial of each visual velocity is plotted for one healthy subject (black line), VS1 (blue line), and VS2 (red line).

Examination of the raw data of the head in space immediately following the platform and visual scene disturbances (Fig. 2) reveals that HS produced a small extensor motion of the head (mean latency = 98 ± 27 ms) which was unaffected by the presence of, or change in velocity of the visual scene. This response was followed by head flexion in HS (mean latency = 359 ± 44 ms) and a gradual return to initial head position.

VS1 and VS2 also produced the initial extensor motion (Fig. 2), but with longer latencies (135 ± 35 ms and 171 ± 37 ms, respectively). The next peak of flexor motion emerged with a considerably greater magnitude in the visually sensitive than in the healthy subjects (Fig. 2). VS1, who reported visual sensitivity but had no clinical vestibular signs, produced steep flexor responses of the head (average latency of 263 ± 72 ms) which would be expected if this individual were counteracting the upward velocities of the optic flow field. The latencies exhibited by VS1 are very consistent with the reported 180–200 ms latencies for visual reaction times [10] with additional time to compensate for the delays required for force production in the system [11]. The magnitude of these responses increased with increasing visual velocity. The flexion responses of the head in VS2, who exhibited subtle clinical signs of a vestibular deficit, were also large and were often followed by a steep extension response previously seen in the unstable head behavior of patients with bilateral labyrinthine deficit [12]. The two subjects that had been previously diagnosed with BPPV (VS3 and VS4) exhibited head kinematics similar to HS.

**Figure 3:**
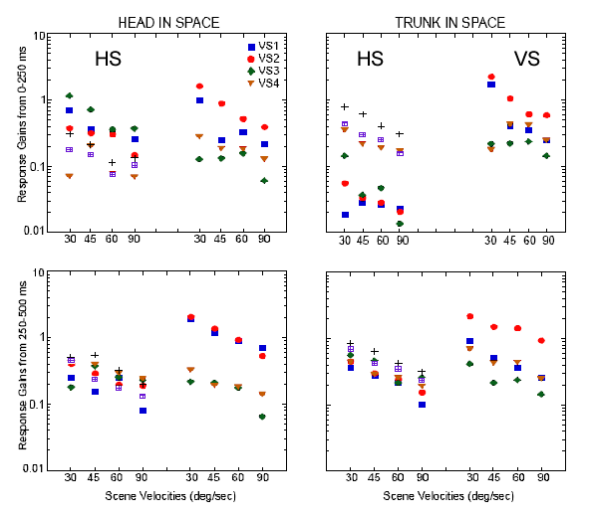
Gain of head (left) and trunk in space (right) angular velocity with respect to scene velocity in the first 250 ms following stimulus onset. Each HS is represented by a different symbol. VS1 is the blue square, VS2 is the red circle, VS3 is the green diamond, and VS4 is the brown triangle.

**Figure 4:**
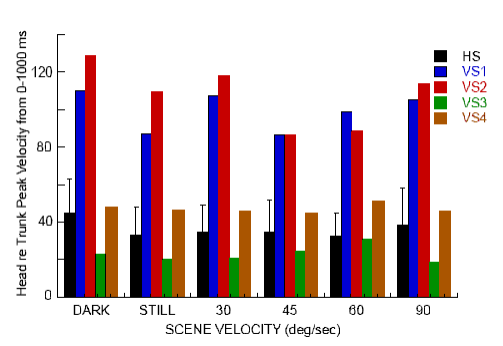
Peak velocity of the head with respect to trunk across the 1000 ms period of the trial at each scene velocity is shown for the mean ± SD across all healthy subjects (black bar), VS1 (blue bar), VS2 (red bar), VS3 (green bar), and VS4 (brown bar).

When the peak velocity of the head and trunk were examined relative to scene velocity there was a slight difference in the gain of the head response between the two groups in the first 250 ms following scene onset that was mostly due to the responses of VS1 (Fig. 3). But, except for VS1 and VS2 at 30/sec, VS subjects exhibited a response gain plateau in the trunk across scene velocities unlike HS subjects that exhibited decreasing trunk gains with increasing scene velocity. In the next 250 ms, there was a distinct separation from all of the other subjects in the response gains of the head for VS1 and VS2. Five of the six HS exhibited response gains between 0.5 and 1 with a downward trend as scene velocity increased. VS3 and VS4 exhibited the same response pattern within the same response bandwidth. VS1 and VS2, however, exhibited head with respect to scene velocity response gains that were greater than one for scene velocities up to 60°/sec. Peak to peak velocities of the head relative to the trunk across the period of the trial further differentiated between the VS subjects (Fig. 4). VS1 and VS2 exhibited much larger motions of the head with respect to the trunk than did the healthy subjects. The 2 subjects with a history of vestibular disorder, however, exhibited peak velocities of head with respect to trunk that were similar to (VS4), or smaller (VS3) than, the HS.

### Out of plane motions of the head

**Figure 5:**
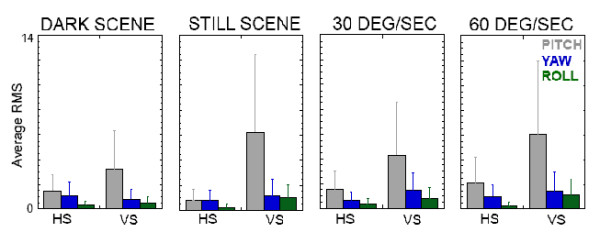
Mean ± SD of the RMS values across 1 sec of head excursions in the pitch (grey), yaw (blue), and roll (green) planes for healthy (HS) and visually sensitive (VS) subjects.

During platform translations, out of plane motions of the head were not apparent in the dark. But RMS values of out of plane motion increased significantly (p < 0.04) in VS subjects when averaged across the conditions where the visual scene was visible (Fig. 5).

## Discussion

Changes in velocity of the optic flow field had no significant effect on EMG responses in the lower limb and trunk. This was not true of the kinematic responses of the head which clearly differed between subjects as visual scene velocity increased. If responses of the head in space were consistent across all scene velocities, then we could assume we were observing responses to the mechanical perturbation of the platform. HS, however, were able to minimize motion of the head in space with a scene reflecting natural vision and with the scene that moved at the same velocity as the physical disturbance, but not when scene the scene was dark or when scene velocity was greater than feedback from the physical disturbance.

Head and trunk angular velocities of the VS subjects oscillated longer and were generally larger than the HS in all planes of motion and at all scene velocities indicating a marginally stable system. Different response latencies and direction of head motion across VS subjects implies that each subject was relying on a different control mechanism. The striking motion of the head in space in the same direction as the optic flow field, and the large short latency response gains in VS1, even when the platform moved at the same velocity as the visual scene, suggest that this subject was unable to integrate the visual and motion feedback. Large response gains of the head that continued across the first 500 ms period of the trial in VS2 suggest that this individual was particularly sensitive to the initial acceleration of the visual stimulus. VS2 also exhibited much longer EMG response latencies and sustained large magnitudes of head with respect to trunk motion when the scene was dark. These results are reminiscent of vestibular deficient individuals who have delayed EMG responses and reduce trunk motion to maintain postural stability, but are unable to stabilize the head in space [11,12]. Clinical testing of this individual did suggest a subtle labyrinthine deficit due to gentamicin toxicity. VS3 and VS4 locked their head to their trunk to reduce head velocity in space from which we infer that they were relying on proprioceptive inputs to stabilize the head, perhaps as a result of learning to adapt to unreliable vestibular inputs when they were diagnosed with BPPV.

## Conclusion

Although each of the patients studied here complained of dizziness and postural disturbances when exposed to busy visual environments, there were disparate effects of combining visual and base of support disturbances on the stabilizing responses of their head and trunk. We believe that these results support the use of virtual reality technology for differential diagnosis and specifically designed interventions for individuals whose chief complaint is sensitivity to visual motion.

## Competing interests

The author(s) declare that they have no competing interests.

## Authors' contributions

EAK designed the study, analyzed and interpreted the data and wrote the paper. JS conducted the experiment and made a substantial contribution to data reduction and analysis. YD developed the model and was involved in interpretation of that data. TCH recruited VS patients and performed the physical examinations. All authors read and approved the final manuscript.

## Supplementary Material

Additional file 1 Click here for file
